# Design aspects for prognostic factor studies

**DOI:** 10.1136/bmjopen-2024-095065

**Published:** 2025-08-31

**Authors:** Peggy Sekula, Inga Steinbrenner, Ulla T Schultheiss, Neus Valveny, Paola Rebora, Susan Halabi, Suzanne M Cadarette, Richard D Riley, Gary S Collins, Willi Sauerbrei, Mitchell H Gail, for topic group 5 of the STRATOS initiative, Mitchell H Gail

**Affiliations:** 1 Institute of Genetic Epidemiology, Faculty of Medicine and Medical Center - University of Freiburg, Freiburg, Germany; 2 Department of Medicine IV - Nephrology and Primary Care, Faculty of Medicine and Medical Center - University of Freiburg, Freiburg, Germany; 3 Synlab MVZ Humangenetik Freiburg GmbH, Freiburg, Germany; 4 Real World Evidence, TFS HealthScience, Barcelona, Spain; 5 Bicocca Bioinformatics, Biostatistics and Bioimaging Centre - B4, School of Medicine and Surgery, University of Milan-Bicocca, Milano, Italy; 6 Unit of Clinical Epidemiology and Biostatistics, IRCCS San Gerardo dei Tintori Foundation, Monza, Lombardia, Italy; 7 Department of Biostatistics and Bioinformatics, Duke University School of Medicine, Durham, North Carolina, USA; 8 Leslie Dan Faculty of Pharmacy & Dalla Lana School of Public Health, University of Toronto, Toronto, Ontario, Canada; 9 School of Health Sciences, College of Medicine and Health, University of Birmingham, Birmingham, UK; 10 National Institute for Health and Care Research (NIHR), Birmingham Biomedical Research Centre, Birmingham, UK; 11 Centre for Statistics in Medicine, UK EQUATOR Centre, Nuffield Department of Orthopaedics, Rheumatology, and Musculoskeletal Sciences, University of Oxford, Oxford, UK; 12 Institute of Medical Biometry and Statistics, Faculty of Medicine and Medical Center - University of Freiburg, Freiburg, Germany; 13 Biostatistics Branch, Division of Cancer Epidemiology and Genetics, National Cancer Institute, Rockville, Maryland, USA

**Keywords:** EPIDEMIOLOGIC STUDIES, EPIDEMIOLOGY, Prognosis, Research Design, STATISTICS & RESEARCH METHODS

## Abstract

Prognostic research is clinically relevant and ultimately facilitates stratified medicine. However, its quality and output are limited. More guidance is needed to improve understanding and thus quality. On behalf of the topic group ‘TG5: study design’ of the STRATOS initiative and for the general readership, this article describes key concepts and issues for prognostic factor studies, a sub-area of prognosis research. After providing a general overview on prognosis research, the article covers aspects such as aims, estimands and designs of prognostic factor studies, highlighting standards and current practice. Focusing on prognostic factor studies that assess a single factor at a time and a binary outcome, this article is complemented by a glossary of terms and a list of general aspects to consider in prognostic factor studies.

## Introduction

 Prognosis is the prediction of the probable course of a disease or its outcome and has been a feature of medicine since the time of Hippocrates.^1^ Accurate prognostic information is valuable for advising patients about likely outcomes and guiding treatment to improve outcomes.^2^ Since treatments can have serious adverse effects, their use may be justified only in patients with a poor prognosis. Consider, for example, chronic kidney disease (CKD), a complex disease of global importance with an increasing estimated prevalence of 8%–16% in the adult population worldwide.^3^ CKD prognosis includes the risk of progression to kidney failure (KF), as well as the risk of mortality and morbidity, which are increased even in persons with mild CKD.^3^ Prognostic instruments to identify CKD patients at increased risk for KF or other serious events could be valuable tools for informing patient counselling, clinical management and healthcare resource planning.^4^

Prognostic information is also useful in the design and analysis of clinical trials.^2 5^ For example, the sample size needed for a clinical trial determining whether a new treatment reduces the rate of cancer recurrence depends on the prognosis of the trial participants, because the power to detect a reduction in the rate of recurrence depends on the number of observed recurrences.^6^,^8^ Prognostic variables are also used to stratify participants of clinical studies into risk groups with comparable outcome probabilities across treatment arms to improve the randomisation process. Also, when adjusting for prognostic variables in the actual analysis, the power to detect genuine treatment effects in a clinical trial can be increased.^9^ Furthermore, prognostic variables are often used as candidates for explaining treatment effect heterogeneity (also known as predictive factors^10^ or treatment-covariate interactions). Both predictive and prognostic factors facilitate stratified medicine.^11^

To improve the prediction of outcomes in diseased patients, single prognostic factors are usually combined in a prognostic model (box 1: prognostic model research).^12^ Such models typically include demographic factors such as sex and age, and disease-specific factors such as estimated glomerular filtration rate for KF or prostate-specific antigen for prostate cancer relapse.^13^,^15^ Examples of prognostic models frequently used are the ‘Nottingham Prognostic Index’ for predicting breast cancer survival or the ‘Corticosteroid Randomisation After Significant Head Injury’ (CRASH) models for the outcome of traumatic brain injury.^16 17^

Box 1 **What is prognosis research?**

**1. Descriptive prognosis research**

Descriptive prognosis research describes the spectrum of possible outcomes and their impact and burden in patients with a specific disease.^31^ This perspective thus provides an overall description of outcomes in populations defined by particular conditions and healthcare settings and forms the rationale for many subsequent studies, such as studies to identify specific prognostic factors and to quantify risks precisely.

**Example:** Keith *et al* described the course of CKD patients in a large cohort with respect to clinical outcomes and comorbidities.^111^

**2. Prognostic factor research (single factor of interest)**

**Figure 1:**
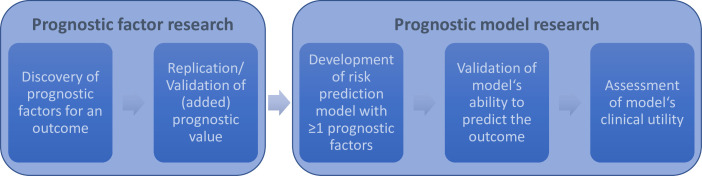
Prognostic factor: illustration of stages in research. See box 1 for more details.

The aim of prognostic factor research (figure 1) is to identify individual factors that enable to predict the patients’ future outcome.^2^ Such factors do not need to be causally related with the outcome to be prognostic. Prognostic factors should be distinguished from ‘predictive factors’ that predict the size of a treatment effect.^10^

To identify novel prognostic factors, recent studies increasingly use laboratory techniques that permit high throughput measurements from samples from large populations and that allow measurement of many features on each sample (‘Omics’ data, eg, genomics data).^112^

Identifying novel factors (discovery) usually starts with tests for an association with an outcome, including their characterisation in patients with and without the outcome. After its discovery, further studies are needed to estimate risk-factor specific disease risk, to assess its value for prognostic risk estimation, and also to validate any findings. Furthermore, development and validation of reliable ways to measure the novel factor might be requested.

**Example:** Figure 1A in Go *et al* illustrates the association of lower estimated glomerular filtration rate (eg, FR; corresponding to a poor kidney function) with increased risk for death.^58^

**3. Prognostic model research (multiple factors of interest)**

While a single factor may have limited ability to predict an outcome, several factors together may increase this ability. Prognostic model research (figure 1) focuses on developing a new model or improving or updating an existing model that incorporates several prognostic factors and yet can be easily applied in daily patient care.^12^ In addition to evaluating and validating the prognostic ability of models, prognostic model research also aims to assess their clinical utility.

**Example**: Tangri *et al* developed a model containing four prognostic factors of CKD progression and kidney failure.^13^

**4. Stratified medicine research**

Since the ultimate goal of prognosis research is to advise patients and guide treatment decisions, the aim of this research is to tailor these decisions using prognostic models in combination with knowledge on factors predicting treatment effects (‘predictive factors*’*) and thereby facilitating stratified medicine.^11^

Even when a prognostic model is available for a specific setting, its ability to predict the outcome accurately is often limited. Consequently, there is a need to identify additional prognostic factors (box 1: prognostic factor research) and subsequently to develop a new or to update an existing prognostic model (figure 1).^2^ Despite some success stories, the impact of prognostic factor research (or biomarker research in general)^18^ has been limited, as too many proposed factors fail further testing and are not usefully incorporated into prognostic models.^419^,^22^ Methodological issues that limit the validity and reproducibility of findings often impede the clinical application of prognostic instruments and prevent consensus on its utility that is usually achieved by combining relevant studies in a systematic review.^519 20 23^,^30^

Various working groups provide guidance and solutions to improve prognosis research. The PROGnosis RESearch Strategy partnership aims to improve all facets of prognosis research (http://prognosisresearch.com),^31 32^ and the STRengthening Analytical Thinking for Observational Studies (STRATOS) offers guidance for observational studies (http://www.stratos-initiative.org/).^33^ STRATOS divides different aspects of observational studies into topic groups (TGs) such as ‘TG1: missing data’, ‘TG5: study design’ or ‘TG8: survival analysis’.

On behalf of the topic group ‘TG5: study design’ of the STRATOS initiative, this paper aims to provide guidance for a non-specialist readership on design issues for prognostic factor studies, using four published studies to illustrate key concepts and issues (table 1).^34^,^38^ For readers less familiar with statistical and other terms, the article is accompanied by a glossary with terms which are italicised in the following text (online supplemental information). In any case, we strongly advise everyone with less statistical expertise to consult researchers with respective expertise (eg, biostatisticians).

**Table 1:** Four published prognostic factor studies

Publication	Albertsen *et al* (1998; 2005)^34 35^	Varlotto *et al* (2008)^36^	Consonni *et al* (2015)^37^	Huzell *et al* (2015)^38^
PMID	9749479; 15 870 412	18 260 087	25 802 059	25 556 354
Research question regarding prognostic factor(s)	*p. 975 Abstract (1998*) ‘To estimate survival based on a competing risk analysis stratified by age at diagnosis and histologic findings for men diagnosed as having clinically localised prostate cancer…’ *p. 2095 Abstract (2005*) ‘To estimate 20-year survival based on a competing risk analysis of men who were diagnosed with clinically localised prostate cancer and treated with… stratified by age at diagnosis and histological findings’.	*p. 1547 Abstract* ‘…to assess whether disease-specific survival (DSS) and overall survival (OS) differed among patients who had N1 and N2 bronchioloalveolar carcinoma (BAC) compared with patients who had non-BAC non-small cell lung cancer (NSCLC)’.	*p. 1 Abstract* ‘…evaluated prognostic factors for recurrences and survival in 2098 lung cancer case patients … conducted survival analyses and estimated absolute risks separately for stage IA to IIIA surgically treated and stage IIIB to IV non–surgically treated patients’.	*p. 505 Abstract* ’…was to study oral contraceptive (OC) use in relation to breast cancer events… in a prospective population-based cohort…’
Disease of interest	Prostate cancer, clinically localised	Non-small cell lung cancer (NSCLC), N1/N2: lymph node status	Lung cancer	Primary breast cancer
Exposure of interest	Age at diagnosis Gleason score	Bronchioloalveolar carcinoma (BAC, binary)	Stage IA–IV treatment surgical (ST), non-surgical (NST)	OC (binary)
Study design/study type	**Registry-based study, retrospective** Connecticut Tumour Registry (CTR) Hospital records from 36 acute care hospitals and 2 Veteran Affairs medical centres	**Registry-based study, retrospective** source: SEER registry data access: 3 May 2006	**Add-on study** ongoing case-control study participants are followed prospectively Lombardia, Italy	**Add-on study** ongoing prospective cohort study start in October 2002 Lund, Sweden
		*p. 1548 Materials and Methods* ‘The case ascertainment rate from the SEER registries has been reported to be 97.5% and is believed to be a general representation of the entire American population’.	*p. 2 Methods* ‘Thus, the enrolled patients were quite representative of all the case patients’.	
Recruitment period	1971–1984	1992–2002	2002–2005	2002–2011
Inclusion and exclusion criteria	**Patients:** with prostate cancer aged 55 to 74 years at diagnosis without metastases	**Patients:** with NSCLC, stages I–IIIB who underwent definitive surgical procedure	**Patients:** with lung cancer diagnosis Apr 2002–Feb 2005 defined catchment area in Lombardia	**Patients:** with primary breast cancer not preoperatively treated without carcinoma in situ without metastatic spread within 3 months of surgery
Groups of comparison, study size	Gleason score: 2–4: n=138; 5: n=118; 6: n=294; 7: n=137; 8–10: n=80 Age at diagnosis: 55–59: n=54; 60–64: n=141; 65–69: n=242; 70–74: n=330	Unmatched data set Group: BAC (n=684) (N1: n=408; N2: 276) Group: non-BAC NSCLC (n=9809) (N1=5626; N2:4183)	Stage IA–IIIA: 1018 (ST n=760, NST n=258) Stage IIIB–IV: 1032 (ST n=61, NST n=971) Stage X: 48 (NST)	Never use (n=276), ever use (n=671) (OC-status missing for one patient) OC use before age 20 (yes - n=305, no - n=643) OC use before first child (yes - n=467, no - n=478)
	**In total:** 767	**In total:** 10 493 (matched set: 646)	**In total:** 2098	**In total:** 948
Follow-up	*1998*: follow-up until Mar 1997, mean follow-up: 8.6 years *2005*: extended follow-up until Dec 1984, median follow-up (range): 24 years (16-33)	Matched pair set: median 29 months	Range of median follow-up by stage and treatment: 0.7–4.1 years, end of study: Dec 2010	Median follow-up 3.03 years (IQR 1.93–5.23)
Outcome(s) of interest	Death, any cause and cause-specific	OS, DSS	OS, recurrence	breast cancer events (eg, local recurrence, distant metastasis)
Statistical methods	Estimation of probability of dying from prostate cancer or other competing causes, regression-based competing risk analysis	Log-rank test, Kaplan-Meier curves	Kaplan-Meier analysis, log-rank test, Cox regression (HR), Estimation of mortality and recurrence rates, Estimation of absolute risks	Kaplan-Meier analysis, Cox regression (HR, adjusted)
Number of events	Until March 1997: 610 deaths Cause of death known for 553 Mar 1997–Dec 1984: 107 further deaths In total: 717 deaths If missing (n=25), cause of death was imputed	*Not reported*	1692 deaths (including 78 from other causes), 633 recurrences (IA–IIIA: 375; IIIB–IV: 258)	100 breast cancer events during follow-up
Sample size or power	*Not reported*	*Not reported*	*Not reported*	*Not reported*
	*p. 957 Introduction (1998*) ‘… and (4) a sample size sufficiently large to permit stratification by the biopsy Gleason score and age at diagnosis…’			*p. 513 Study limitations ‘*The median follow-up time was relatively short’.
Validation/replication	*Not reported*	*Not reported*	*Not reported*	*Not reported*
				*p. 513 Study limitations* *‘*Since this is an exploratory study…the results need to be confirmed in an independent material’.

In the remainder of this paper, we focus on studies assessing the *prognostic value* of a single factor at a time for a prespecified *outcome*. Moreover, we limit our attention to *binary outcomes* such as the occurrence of KF or death in CKD patients by a particular time-point.

## Aims of prognostic factor studies

Any *prognostic factor* study should be conducted specifically in a sample of the diseased population for which prognosis is intended (so-called target population). While the general aim of *prognostic factor* studies is to identify individual factors that predict patients’ future *outcome* (box 1), the aim of a single *prognostic factor* study is far more specific and depends on the stage of research with respect to the factor of interest. Consonni *et al*, for example, aimed to estimate risk for lung cancer recurrence depending on the stage and treatment of the disease (table 1).^37^ To provide a brief overview:

*Discovery* studies aim to identify novel factor(s) by screening one or more factors for an association with the *outcome*. Recent studies increasingly use laboratory techniques that permit high throughput measurements from samples from large populations and that allow measurement of many features on each sample (high-dimensional ‘Omics’ data, eg, genomics data).^39^ A factor that is associated with the *outcome* (so-called *risk factor*) can be useful for prediction whether or not it is causally related to the *outcome* event occurrence.^40^

However, establishing an association of the factor with the *outcome* is not sufficient in itself to demonstrate its usefulness in informing prognosis (ie, in estimating individual *outcome* risk).^41 42^ In addition, the *prognostic value* of a factor and eventually its *clinical utility* depends on the prevalence/variability of the factor itself as well as on the incidence of the *outcome*.^43^ For example, a rare *risk factor* may have little use in prognosis. Another aim of the *prognostic factor* study is thus to assess the *prognostic value* of a factor, especially in addition to established *prognostic factors* (so-called added or incremental *prognostic value*).

Any finding of a *prognostic factor* study should be subjected to internal and external *validation*.^44 45^ In contrast to *internal validation* of results, the *external validation* uses independent data (eg, obtained by a different study group) to check on the validity of observed results. Such attempts may even justify a separate study, in which the *generalisability* of the results could also be evaluated. An example for such a study is reported by Tangri *et al* evaluating the KF risk equation in different geographic regions and different populations and presenting results from combined individual participant data (IPD) in a meta-analysis.^46 47^

All in all, more than one study is usually required to establish a new *prognostic factor*. Indeed, a systematic review of published studies together with a meta-analysis is a state-of-the-art method for synthesising evidence.^30^ Although more complex, a meta-analysis using IPD is preferable because it may avoid problems that can arise when combining summary statistics.^30 47^

Only when a novel factor has proven to be prognostic and can be measured reproducibly, its inclusion into a prognostic model for clinical application may be considered (box 1: prognostic model research).^12 48^ For novel measurements, even additional aspects need to be considered, such as the development of reliable assays to measure them for research purposes, but ultimately also for clinical application.

## Data and measurements

When designing a *prognostic factor* study, it is critical to identify the data needed to answer the research question. At a minimum, accurate information on the factor and the *outcome* of interest is needed, as well as on previously established *risk* and *prognostic factors* of the *outcome of interest*. Clear definitions and well-defined data collection procedures are required for each data item. Standard operating procedures for data collection and quality control can reduce problems with data such as systematic errors in measurements, and thereby ensure high quality.^49^

*Prognostic factor* studies require details of the measurement of the factor(s) of interest, which should be done at least once at the beginning of follow-up (baseline). To further improve prediction of *outcome*, longitudinal measurements of certain factors (ie, serial measurements) may be useful, as has been, for example, recommended for carcinoembryonic antigen (CEA) for colorectal cancer.^50^ If one only takes CEA measurements when a patient has abdominal pain following treatment for colorectal cancer, one will overestimate the association of elevated CEA with colorectal cancer recurrence (see chapter on ‘Some Statistical Methods for Immunodiagnostic Cancer Tests’ in Herberman and Mercer).^51^ Longitudinal measurements may allow dynamic predictions.^52^

For some *prognostic factors*, such as serum creatinine, high-quality standardised measurements are available which should be used. However, standardised procedures may not be available for novel factors of interest, which jeopardises the reproducibility of findings from a single study and comparability across studies.^21 53 54^ While the development of a standardised measurement procedure may come later, the basic measurement techniques should be clearly defined *a priori* to promote the internal validity of the measurements.^10 53 55^ In particular, laboratory quality measures (eg, coefficient of variation) and basic description of observed measurements should be provided for any novel factor. A pilot study is useful to demonstrate analytical validity, reproducibility and robustness of measurements.^53 54 56^

All data should be recorded on the original scale. Categorisation of *continuous* factors should be avoided when collecting data to prevent loss of information.^25 57^ To avoid mistakes, composite measures that are derived from several single measurements (eg, body mass index is derived from height and weight) should be calculated using a programmed algorithm after data collection from the primary data. Measurements that rely on subjective assessments, such as the rating of staining intensity on a slide or the estimation of the percentage of fat tissue on a mammogram, are subject to rater variability. Using more than one rater, each blinded to the source of the biospecimen, can reduce misclassification and bias.^53^

## The choice of outcome

The choice of study endpoint (*outcome*) is critical for answering the research question and other aspects of the study design. Some endpoints are important for defining pathophysiology, while others are chosen for their relevance to clinical management.

As an example, consider the endpoint all-cause mortality. This endpoint is clearly clinically relevant. However, due to the diversity of causes of death, the *prognostic value* of predicting all-cause mortality may not be as useful for clinical care as cause-specific mortality.^26^ CKD patients, for example, suffer from a higher overall risk of death.^3^ However, CKD patients often die from causes other than untreated KF, such as cardiovascular events.^58^ Depending on the research question, endpoints such as KF requiring kidney replacement therapy or cardiovascular mortality might be more meaningful.

In the four examples in table 1, the authors considered at least one disease-specific *outcome* such as disease recurrences or disease-specific survival, which are events observed over several years.^34^,^38^ For more information on the definition of clinical *outcomes*, see also Box 3 in the REMARK guidelines paper.^59^

## How to specify and summarise the outcome appropriately?

While the validity and quality control of *outcome* data are just as important as with other data, another important aspect relates to the specification of exactly how the *outcome* is to be measured and analysed. For *outcomes* such as the occurrence of KF or death in CKD patients, data can be understood as a *binary* event which occurred within a defined follow-up period (eg, 1 year). Alternatively, the *time-to-event* can be in the focus of a study.^60^ *Time-to-event* information includes not only whether and when an event (eg, KF) has occurred in a given period after the patient entered the study (time 0=baseline), but also the time of study closure (so-called administrative censoring), or whether and when a specific participant was lost to follow-up. In many settings, other events (‘competing risks’) also occur in the course of a study that prevent the occurrence of the event of interest.^61^ In a study with the *outcome* KF, for example, a competing event would be the death of individuals from non-KF causes such as myocardial infarction.

To summarise *outcome* data, an important prognostic quantity is the probability that the event of interest will occur in a defined time period, such as within 5 years of diagnosis or study inclusion (so-called ‘*pure risk*’). Often, to account for administrative censoring or loss to follow-up, this probability is estimated from survival curves (eg, using Kaplan-Meier estimator).^60^ Survival curves are functions of time that describe the probability of not having the event of interest at or before a time *t*. Since competing risks reduce the chance that the event of interest will occur, the more clinically relevant type of risk is called *absolute risk*.^61^,^63^ The differentiation between *absolute* and *pure risks* is especially important for long-term prognosis, whereas they are often similar over short time intervals. Special methods are available to compute *absolute risk* (eg, estimating cumulative incidence function).^61 62^

Instead of using *estimates* of *pure* or *absolute risk*, investigators sometimes use median survival or restricted mean survival to measure prognosis.^8^ However, because survival times (or more generally *times-to-events*) are variable, even for individuals with a similar underlying risk profile, the cumulative probability of the occurrence of the *outcome* to a given time point (ie, *absolute risk*) may be more valuable for guiding clinical management than a statement, for example, about median survival.^64^

## What is the estimand of interest from prognostic factor studies?

Before the design of a *prognostic factor* study can be determined, the measure of interest (so-called *estimand*) one wants to estimate in the study with a given aim needs to be specified.

For a *prognostic factor* study with the aim of identifying the association between the potential *prognostic factor* and the *outcome* of interest, useful measures of association are *relative odds* (OR), *relative risks* (*risk ratio*) or *relative hazards* (HR).^62 65^ The interest may lie on the estimation of associations in the absence (so-called unadjusted) or in the presence (so-called adjusted) of known *prognostic factors*.

If the aim of the project is to assess the *prognostic value* of a factor by estimating *pure* or *absolute risks*, other measures might be used to evaluate the performance of risk estimations with respect to *calibration* (using *calibration* plots), *discrimination* (measured by the *area under the receiver operating characteristic curve*) or mean squared *prediction error* (called the Brier score for *binary outcomes*).^66^,^70^ These measures can also be used to assess the added value of a *prognostic factor* by comparing corresponding measures derived from a model that includes only known *prognostic factors* with a model that additionally includes the factor of interest.^66 68 70^ Further, measures such as *R²* can also be used to assess model’s goodness-of-fit to describe the *outcome*.^66 70^

## Study designs for prognostic factor studies

After clarification of the study aim and the *estimand* one seeks to estimate, an appropriate design can be chosen taking into account the medical context of the disease (eg, treatments).

Because factors of interest are usually not subject to experimental control as they are either non-modifiable (eg, genetic predisposition) or *risk factors* with potential detrimental health effects and thus unethical to administer to participants of a study (eg, smoking), *prognostic factor* research is mainly done on observational data.^65^

If the purpose of the study is simply to determine whether a factor is associated with an *outcome*, a *case-control study* design may be appropriate.^65^ Comparison of factor’s distributions between individuals selected from the target population by *outcome* status (presence, cases; absence, controls) leads to estimates of the *relative odds* of disease per unit increase in the *risk factor* (if *continuous*) or versus the reference category (if *binary* or *categorical*). Its calculation from case-control data theoretically corresponds to the *relative odds* of the *outcome* that can alternatively be obtained from a *cohort study*.^71^ *Case-control studies* have limitations.^65 71^ Major issues of case-control designs in *prognostic factor* research include ensuring that the prognostic variables were reliably measured and correspond to the start point at which prognostic information was required. Accounting for censored observations is also problematic.

**Figure 2:**
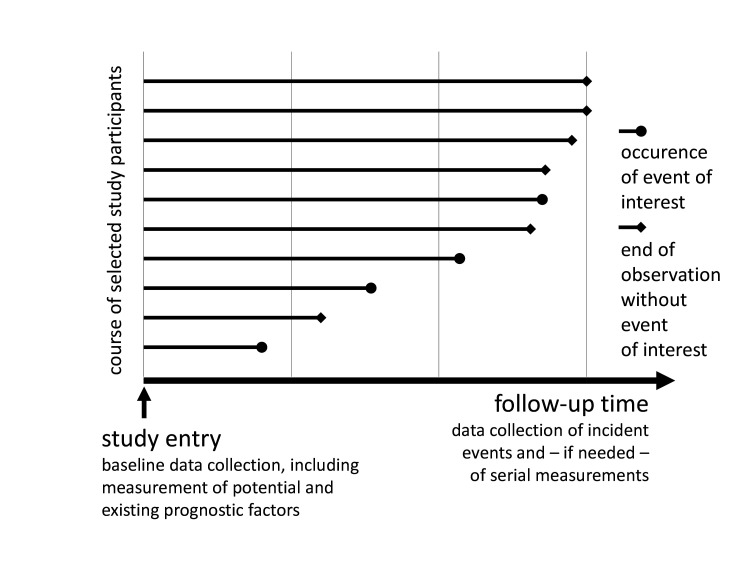
Conceptual illustration of a cohort study for use in prognostic factor research. In the case of a *prospective cohort study*, the study entry lies in the present, whereas in a *retrospective cohort study*, the cohort is assembled retrospectively from information collected in the past. Of note, the calendar date of study entry of the participants does not necessarily have to be the same. Nevertheless, participants in both types of studies are followed as time goes on starting at the time of inclusion. For retrospective cohort studies, follow-up data may still be available from the past, but data collection may also continue in the future. An event of interest may be any prespecified outcome such as kidney failure in patients with chronic kidney disease. End of observation may include censoring due to administrative closure of the study or the loss of contact with some participants (lost to follow-up) or the occurrence of a competing event such as death from causes other than kidney failure.

Alternatively, but also if the purpose of the study is to estimate *absolute* or *pure risk*, the gold-standard design is a *prospective cohort study* (figure 2).^62 65^ For this purpose, participants are recruited from the target population for whom data are available on factors(s) that were measured at or before the starting point of interest for prognostication. Although restrictive study inclusion and exclusion criteria may yield a homogeneous study cohort useful for identifying new *prognostic factors*, a more representative cohort can yield risk predictions that are more accurate for the target population.^72^ Of advantage, *outcomes* can be completely and accurately assessed during the follow-up of this cohort. Important aspects to consider when designing a cohort study are the timing of visits and the length of follow-up. The follow-up should be conducted on a fixed schedule for all study participants, regardless of their clinical status, to avoid selection bias or informative missing data, and to mirror clinical practice.^53 73^ In the end, a *cohort study* yields information on measures of association such as *relative odds* or *relative hazards* as well as on *pure* and *absolute risks*.

Overall, an advantage of a *prospective cohort study* tailored to the purpose of *prognostic factor* research (ie, data collected specifically for the condition and *outcomes* of interest) is that one can collect high-quality data including previously established *prognostic factors* and treatment. However, a *prospective cohort study* is often costly and takes a long time to observe a sufficient number of events of interest during the follow-up.^10 53 65^

### Using already available data and biospecimens: pros and cons

To avoid costs and time issues of de novo *prospective cohort studies*, researchers have taken advantage of already collected data and biospecimens in the past.^7 24 74^

*Prognostic factor* research can also be conducted as an add-on project to an existing *prospective cohort study* whose study population resembles the target population of interest.^53 75^ The existing study may have standardised procedures for collection of baseline data and biospecimens and for *outcome* ascertainment, and it may be set up to support add-on studies, saving time and expense. The German Chronic Kidney Disease study is an example of an ongoing prospective observational study of patients with moderately reduced kidney function at baseline providing a supportive structure for add-on projects.^76^

If required data are available on all members of the cohort, standard methods can be used.^8 60 62^ However, required data are sometimes only available for a subcohort when, for example, it is too expensive to analyse biospecimens from all members of the cohort to obtain measurements on the factor of interest. Fortunately, one can still estimate *absolute* or *pure risk* by obtaining such factor data from cases and a subsample of non-cases using a *nested case-control* or *case-cohort* design.^62 65^ These subsampling strategies can greatly reduce the cost of prognosis research but still require other baseline data from all cohort members.

Randomised clinical trials include prospective cohorts that are also potentially useful for observational prognosis research. Their specific advantages are that the initial treatment regimens are known, the population is well defined and the baseline data and follow-up data are usually of high quality.^75^ However, *estimates* of *pure* or *absolute risk* from clinical trial populations may not be *generalisable* to the target population of interest if the clinical trial population is highly restricted by eligibility criteria or if the follow-up procedures for ascertaining events differ from those in common use in the target population.

Other secondary data sources, like registries, databases, electronic health records (EHRs) or biospecimen repositories may also provide a basis to retrospectively assemble a cohort (often called: *retrospective cohort study*; figure 2) for prognosis research.^53^ Some sources are dedicated to specific diseases or groups of diseases, such as the German Centre for Cancer Registry Data, which obtains data from all cancer patients in Germany (https://www.krebsdaten.de/Krebs/EN/Home/homepage_node.html). Other sources like EHRs usually cover a larger population but may not be large enough to include enough people with the disease of interest.

While secondary data sources are very convenient, they may have disadvantages—especially in the case of sources like registries, databases or EHRs.^5 10 19 24 53^ Among others, the source population for people with the disease of interest of a registry, database or EHRs can be less well-defined than in studies with specific entry criteria and thus limit its representativeness for the population that is targeted in the *prognostic factor* study. Ideally, the representativeness of any secondary data source is assessed before using it for prognosis research. In addition, the extent and definitions, as well as the timing and quality of data covered in these sources, might be limited, less complete and not rectifiable.^7 72^

The four studies highlighted in table 1 are examples of studies leveraging already collected data. Varlotto *et al* used data from an established cancer registry in the USA (Surveillance, Epidemiology, and End Results Programme database, https://seer.cancer.gov/), for which a case ascertainment rate of 97.5% in detection areas was reported.^36^ The authors thus provided evidence for the representativeness of underlying data.

Albertsen *et al* ^34 35^ also present a registry-based study (table 1) that identified patients with prostate cancer between 1971 and 1984 in the Connecticut Tumor Registry. Registered patients had been followed according to registry protocols to determine the date and cause of death.^34^ Hospital records of eligible patients were additionally reviewed to successfully online supplemental information available in the registry.

In summary, there are clear advantages to add-on studies by leveraging previously gathered baseline data, biospecimens and follow-up information. However, these advantages may not outweigh limitations of these data sources. Thus, before deciding to base prognosis research on an existing study or registry, one must evaluate the appropriateness of the data source and consider its limitations. Disadvantages may be acceptable in early stages of *prognostic factor* research (eg, *discovery*; figure 1), but a *prospective cohort study* tailored to the target population is preferred in advanced *stages of research* (box 1).

## Statistical methods

Depending on study aim, *estimand* of interest and design, the statistical analysis should be planned accordingly.

In *discovery* studies with a case-control design, for example, standard *statistical tests* might be used respectively to compare the distribution of a *continuous* factor or *binary* factor in cases and controls. However, fitting *regression models* to case-control but also to cohort data is preferred to account for other known *prognostic factors* which traditionally are known as confounders in observational studies, but here are merely other *prognostic factors*.^8 71 77^ While the estimation of unadjusted associations is valuable to describe the raw dependency of the factor with the *outcome*, adjustment for known prognostic factors may indicate whether the factor carries additional independent information about the *outcome*. This adjustment for existing *prognostic factors* allows the researcher to examine the bespoke factor’s added prognostic value, over and above that from the existing *prognostic factors*. Various *regression models* are available. For example:

A logistic *regression model*, for example, can be used to describe the (adjusted) association of a factor in terms of the *relative odds* of the *outcome* with and without the factor in a *case-control study*.^71^ When censoring, loss to follow-up and competing events are negligible in a cohort study, as in cohort studies with short follow-up times (eg, a study of death from heart disease within 1 month of a myocardial infarction), a logistic *regression model* can also be used to estimate (adjusted) ORs.^63 71^ For *time-to-event* data of a *cohort study*, *survival methods* (eg, Cox proportional hazard *regression model*) can be used to estimate (adjusted) associations (*relative hazards*).^60^

*Regression models* allow for the derivation of *absolute risks* of individuals, necessary when aiming to assess the *prognostic value* of a factor.^62 63^ A necessary ingredient is the baseline risk of the *outcome*. In contrast to cohort studies, *case-control studies* do not allow their direct estimation, so that the information must be obtained from published data.^78^ However, when transferring back to the *absolute risk* scale, care should be taken to account for the competing event to reflect a real world situation where the competing event may occur.^61 63^

Furthermore, assumptions underlying every *regression model* (eg, Cox model: proportional hazard assumption^60^) need to be checked. Usual assumptions of linearity and additivity refer to how variables are selected for inclusion in the model.^79^ This includes decisions on how *categorical* variables with three or more levels are coded and whether a *continuous* variable can be included on its original scale.^80^ A *continuous* variable should not be categorised, unless for biological reasons.^25 57 63^

Published *prognostic factor* studies report a mixture of (adjusted) effect sizes in terms of *odds*, *risk* or *hazard ratios*, and *pure/absolute risks* at particular time points. Consider the four examples of *prognostic factor* studies in table 1. Huzell *et al*, for example, assessed the effect of oral contraceptive use (eg, use before age 20 years—no/yes) on the prognosis of breast cancer patients and reported, among others, measures of association (*hazard ratios*) with breast cancer events (eg, recurrence) using a Cox proportional hazard *regression model*.^38^ In comparison, Albertsen *et al* estimated survival in male patients with prostate cancer depending on age at diagnosis and histological findings.^34 35^ By differentiation of the *outcome* of interest (death from prostate cancer) from death from other causes (competing risk), the authors provide *estimates* of cumulative mortality (*absolute risks*) across various ages and histological findings as illustrated in figure 1 of Albertsen *et al*.^34^

In *discovery* studies with high-dimensional omics data, several approaches are available. Researchers may wish to evaluate each omics feature separately. In this situation, there is not much difference in the procedure compared with a study that evaluates only one factor—apart from the burden of evaluating so many features in parallel. To avoid too many falsely positive results (ie, inflated type 1 error), methods to adapt the significance threshold have become standard.^81^,^83^ Another approach to deal with many features is to reduce dimensionality by either selecting a subset of features or by defining substitutes supposedly carrying the relevant information of whole omics data. Regularised/penalised regression methods (eg, Lasso regression) and machine learning/artificial intelligence approaches (eg, random forest, neural networks) are methods of choice.^79 84 85^

More detailed literature on statistical methods, their application and recommendations can be found elsewhere.^8 32 59 62 63 71 86^ Of note, a special procedure is required when aiming to validate the *prognostic value* of a factor or model.^87^

## Sample size and power of a prognostic factor study

Assuring adequate power is one of the most important features for the success of a *prognostic factor* study.^2 7 53^ For a *prospective cohort study*, the investigator may be able to estimate sample size to ensure adequate statistical power, whereas the sample size is usually fixed in retrospectively assembled studies. The power of such a study should be assessed instead. Because the number of events reflects the effective sample size driving the power of *statistical tests* to compare proportions and of *time-to-event* analyses, considerations of sample size and power are particularly relevant for less common *outcomes*.^6^,^8^

Standard methods to calculate the sample size or evaluate power are available for some aims of *prognostic factor* research.^88^,^92^ For a *binary* factor and an unadjusted effect of interest, for example, the calculations are similar to those for a clinical trial. Requested inputs are the *relative risk* between groups with and without factor and the prevalence of the factor.^90^ However, where an adjusted effect is required, the sample size needs to account for the correlation with the other factors, which usually inflates the sample size needed.

Since prior knowledge is always required for the calculation of sample size or power, a search of published literature is needed. The result of the calculations can then indicate whether the study is feasible and worth pursuing. If the sample size or statistical power is inadequate to meet the aims of the study, the study should not proceed without revision of the design, because otherwise the study is likely to produce misleading or useless results and a waste of biospecimens and investigator resources. Waste of valuable biospecimens raises ethical concerns.^23 88 93^

Researchers could consider increasing the sample size by collaborating with researchers at other institutions. Collaborative projects, however, require additional planning for data harmonisation and statistical analysis. Results from such endeavours could be reported from the analysis of combined IPD (preferred) or meta-analysis combining summary statistics from contributing institutions.^30 47^ Such a collaboration would also have the advantage of (considerably) reducing problems of heterogeneity which frequently arise due to various differences between studies and their analyses (eg, variables used for adjustment).^30 94^

In practice, sample size considerations are rarely reported in *prognostic factor* research, and many such studies, especially studies leveraging existing data, are too small.^87 95 96^ The four publications in table 1 did not report sample size or power calculations.^34^,^38^ Some would argue that after the study is completed, quantities such as confidence intervals for *relative hazards* or *absolute risks* indicate whether the sample size was sufficient. However, a significant result might be a false positive, making judgements from such quantities vulnerable.^97 98^ In addition, low power also results in more incorrect non-rejection of null hypotheses (false negative). Therefore, considerations regarding the power of a study should be made before starting the study and then also reported.^90^

## Study protocol and reporting

A carefully written protocol is an essential element of a good design of a *prognostic factor* study.^2 5 26 99^ Writing the protocol by the study team helps assure that the aims, definitions and methods are understood and agreed to.

**Figure 3:**
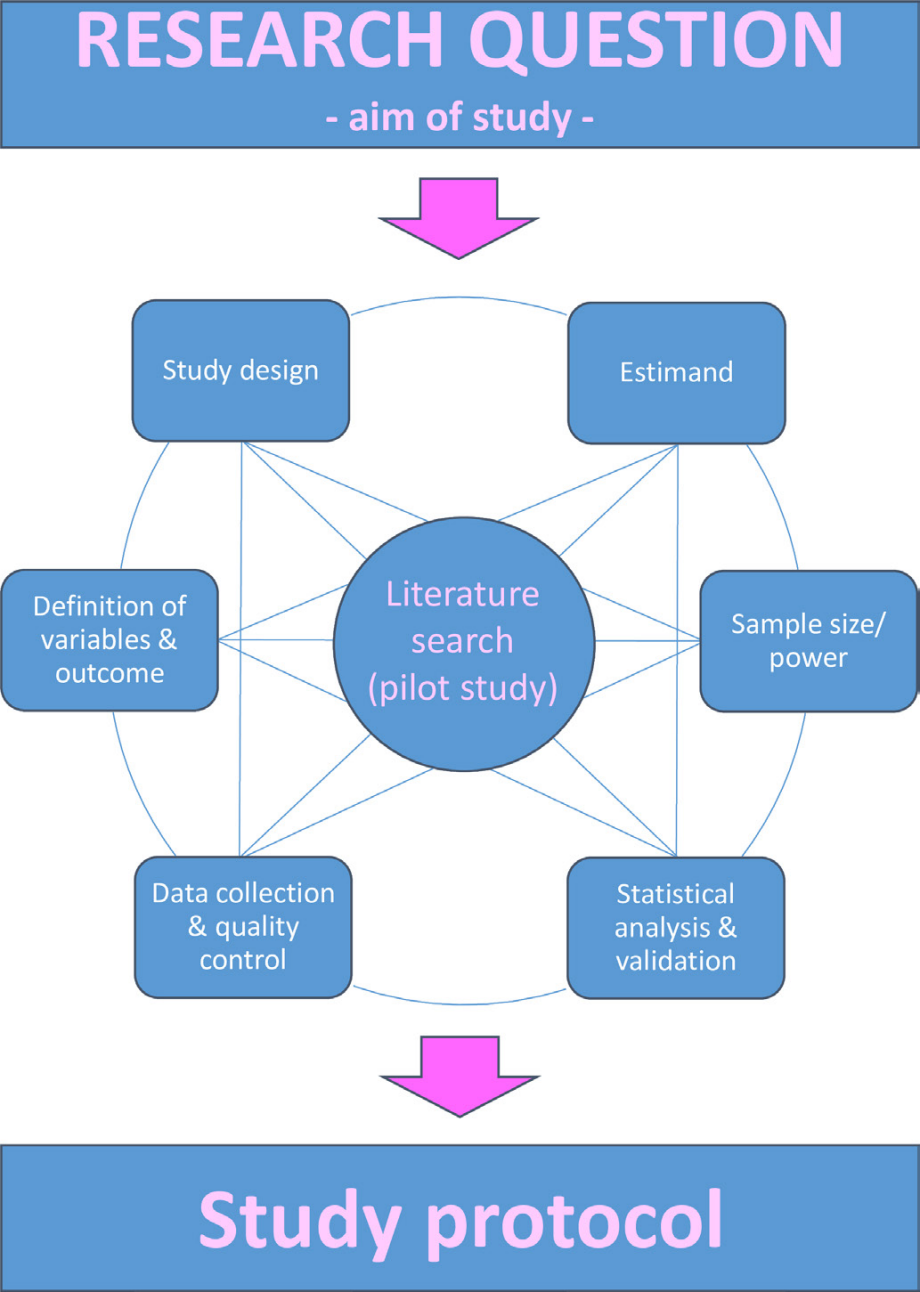
Conceptual illustration of the inter-related aspects in designing a prognostic factor study. The figure attempts to illustrate aspects involved and their dependencies when designing a prognostic factor study. See table 2 for more details on single aspects.

Templates for different study types are available, such as the harmonised protocol template of the International Society for Pharmacoepidemiology and the International Society for Pharmacoeconomics and Outcomes Research.^39^ They share basic elements. In addition to aspects discussed in this article, background information justifying the necessity of the study and its aims is also required, which can be obtained through a literature review. For the convenience of readers, we assembled aspects and questions to address when designing a *prognostic factor* study in table 2 that may even serve as a guideline or check list. In addition, Figure 3 illustrates the complexity of the process when designing a *prognostic factor* study, which is due to the dependencies of individual aspects. Failures in design or measurements are often irreparable by statistical analysis.

**Table 2:** List of general aspects to consider when designing a study for prognostic factor research

A clear definition of the research goals and objectives is informed by:	
Task	Aspects to consider
Literature search	What information is already available? What is the status/stage of research? What is known about the factors to be studied (eg, distribution, variability)? Which outcome(s) are clinically relevant? What are the prevalences or incidences of the index disease and outcomes? Are there other factors that influence outcomes (eg, treatment, known prognostic factors)? What are major limitations of past studies? Are there data sources that might be used?
Estimand	What is the measure of interest? With respect to stage of research and aim of intended study, discuss the use of relative measures (measures of association) versus absolute measures allowing to evaluate prognostic value of a factor or model.
Definition of population and major variables	What is the target population of interest? Provide unambiguous definitions of the index disease, exposure(s) and outcome(s). Compare definitions applied in past studies for consistency with definitions in the planned study. Define the scale on which variables should be collected. Specify variables that need to be derived from collected data.
Selection of the appropriate study design	Discuss advantages and limitations of available data sources to choose one that best meets the aims and design of the new study (eg, resemblance to target population, definition, quality and completeness of data on key variables, sample size). Define a study design appropriate for the stage of research. Choose inclusion and exclusion criteria that meet study aims without threatening generalisability.
Study size/ power	Define sample size and length of follow-up to yield the number of events needed for adequate power. Account for loss of patients during follow-up in estimating required number of events. If the available sample size is fixed, discuss power to see if the study is feasible. If the sample size and power are not sufficient, consider a multicentre approach.
Data and data collection	Define data collection procedures to promote accurate, reproducible, complete data, especially for the key variables. Develop procedures to detect errors in the data and means to correct them. Consider collecting data on other variables of interest, such as treatment, known confounding factors and known prognostic factors. For novel measurements, ascertain quality of measurements when no report exists.
Study protocol	Compile a carefully written protocol describing rationale, aims and the items above. Describe measures of quality control and initial data analysis of collected data. Present statistical analyses tailored to study objective. Include sensitivity analyses, as needed. *Recommended:* registration of the study protocol.
Additional quality control checks	Is prior knowledge sufficient to justify the study aims and design? Are data on key variables accurate and reproducible, and is there data on the validity of measurements on novel variables to be studied as prognostic factors? Is there a plan to train study personnel to ensure quality and completeness of study data? Are serial samples for prognostic factor measurements obtained independently of the health status of the participant? How will completeness of data collection and follow-up be promoted and assessed? Discuss options for external validation of findings.
Listed aspects need to be considered together as they are not independent of each other and may have influence on subsequent decisions. If any aspect cannot be addressed sufficiently, a pilot study or simulation study might be useful to detect problems and missing information, and thereby overcome issues before launching the main study. Moreover, as a *general recommendation*: Consult an experienced biostatistician when designing a study!	

A protocol not only serves as a guide to the conduct of the study, but it is also useful for communicating the importance and quality of the study to potential funders and as a point of reference when writing publications based on the study. Due to poor reporting of study results in the past,^100 101^ reporting guidelines for different types of studies have been developed and can be found at the homepage of Equator Network (https://www.equator-network.org/). The reporting recommendations for tumour marker prognostic studies (REMARK) are highly pertinent to *prognostic factor* research.^102^ Another pertinent reporting guideline is the TRIPOD-AI guideline.^103^ Although its focus is more on prognostic model research than on *prognostic factor* research, many of the listed aspects are relevant to both and even provide important information on the *validation* of results. Detailed explanations and examples have been provided for several reporting guidelines in separate publications (‘Explanation and Elaboration’), including for REMARK and TRIPOD.^59 104^ Although the primary purpose of reporting guidelines is to educate about good reporting, these papers also provide a valuable reference for the many aspects that need to be considered when designing, conducting and analysing studies.

To improve design, methods and reporting in *prognostic factor* research and also to facilitate systematic review on *prognostic factors*, registration in a public database has been advocated.^510 26 99 105^,^107^ Currently, registration is mandatory for clinical trials and post-authorisation safety studies with marketed drugs, but only recommended for other observational studies.

## Summary

We have introduced the general researcher to elements of *prognostic factor* research, focusing on the evaluation of a single factor at a time with a *binary* (including *time-to-event*) *outcome* to illustrate design and methodological principles. While there are numerous other settings and aspects to consider when designing a *prognostic factor* study, this manuscript cannot address them all. For instance, we did not consider *continuous outcome* measures such as the decline in eg, FR, a state-of-the-art *outcome* measure of kidney function.^14^ Also, while aspects were discussed in the context of prospective cohorts with some extension to retrospectively assembled cohorts, other designs (eg, *nested case-control studies*) and data sources (eg, EHR data) may require additional considerations of other aspects. To cover some of the many other and related aspects of prognosis research, it is recommended that the reader consult literature on the development, *validation* and use of prognostic models.^8 32 62 63^ The *external validation* of any result such as the performance of a *prognostic factor* or model and the systematic review of literature is of utmost importance to eventually reach a final conclusion on the *prognostic value* of a factor or model.^30 87^

Overall, shortcomings in design and methodology lead to wasted efforts and resources and partly explain why only a small proportion of new *prognostic factors* is translated into clinically useful information, eg, as part of prognostic models.^22 27 108^ More time should be invested in designing a *prognostic factor* study to avoid problems that cannot otherwise be resolved in the statistical analysis. Improvements will, however, require the combined efforts of researchers and other stakeholders.^22108^,^110^ Although scientific intuition may lead to promising *prognostic factors*, attention to methodology will be a key element for successful translation of results. Indeed, van Calster *et al* argue ‘that the core problem is a paradox: methodology, the very backbone of science, remains overly trivialised by the scientific community that funds, undertakes and reports (pre)clinical research’.^106^

Finally, it must also be acknowledged that there is not a complete solution or recommendation for all methodological aspects in *prognostic factor* research (eg, for prioritising factors in an *external validation*). With the emergence of new data sources (eg, EHR) and methods (eg, measurement techniques), experts in the field are asked to continue to strive for solutions and recommendations for old and new problems.

We hope that this introduction to *prognostic factor* research will raise awareness of the need for high-quality studies and of potential methodological issues that could be addressed at the design stage and thus avoided. For support, information included in display items will provide some additional guidance. We encourage researchers planning prognosis research to consult an experienced biostatistician early and regularly.

## Supplementary material

10.1136/bmjopen-2024-095065 online supplemental file 1
