# Supplementary material for: Design aspects for prognostic factor studies
Source: BMJ Open. 2025 Aug 31;15(8):e095065. doi: 10.1136/bmjopen-2024-095065 (PMC12406931; doi:10.1136/bmjopen-2024-095065)
Supplement: online supplemental file 1 [file bmjopen-15-8-s001.pdf]

Design Aspects for Prognostic Factor Studies

Peggy Sekula, Inga Steinbrenner, Ulla T. Schultheiss, Neus Valveny, Paola Rebora, Susan Halabi, Suzanne M. Cadarette, Richard D. Riley, Gary S. Collins, Willi Sauerbrei, Mitchell H. Gail, for topic group 5 of the STRATOS initiative

Supplemental Information: Glossary – Terms and concepts in the context of prognostic factor research

Term	Description
**Stage of research**	In prognostic factor research, a new factor requires several stages of research before it can become clinically useful. Initial discovery of an association with outcome, confirmation of the association, incorporation into a risk model, validation of the risk model, assessment of the added value of the new risk model, and if warranted, assessment of clinical utility are examples of these stages. See also **Box 1**.
· Exploration / Discovery	An exploratory study attempts to discover novel factors that are associated with outcomes in patients with a specific disease. Further studies are needed to confirm the association and determine whether the factors discovered can usefully contribute to risk models and to assist in clinical management.
· Replication / Confirmation	Estimates of associations with outcomes or of the performance of risk models that incorporate a prognostic factor require confirmation, ideally in independent data to get unbiased estimates and to assess how transportable the results are in various settings.
· Validation and Generalizability	In order to assess validity of results on, for example, the prognostic value of a factor or model, validation is needed. There are two flavors of validation:<br>***Internal validation:*** Evaluation of results (e.g., the performance of a prognostic model) on the SAME population on which the result was obtained. A standard approach is to fit the prognostic model in part of the data (training data) and test its performance in other "left out" part of the data (test data). The repetition of this procedure of splitting (bootstrapping, cross-validation) can give a more realistic estimates of performance as compared with the testing in the same data used to build the model.<br>***External validation:*** Evaluation of results (e.g., the performance of a prognostic model) in a sample INDEPENDENT of data (including data used for internal validation) used to derive results. Ideally, data obtained through a different study group should be used for this purpose. Even more rigorous external validation could be reached when data come from different geographical location and time period.<br>***Generalizability:*** Generalizability refers to the verification of results in different settings such as in a cohort from a different geographical location, age group or ethnicity and is an aim of external validation.
**Factor**	Factors are variables reflecting an individual's personal characteristic or surroundings. Factors include biological measures (e.g., body height, gene expression), demographic characteristics (e.g., age), health conditions (e.g., presence of diabetes, prior treatments), life-style characteristics (e.g., smoking), or environmental characteristics (e.g., air pollution).

· Biomarker	A biomarker is a biological characteristic of an individual that is objectively measured. Biomarkers may reflect normal biological processes (e.g., systolic blood pressure), genetic profiles (e.g., HER2 negative), pathogenic processes (e.g., low CD4 counts), or pharmacological responses to a therapeutic intervention.[1]
· Risk factor	A risk factor is a factor that increases the likelihood of developing a disease or a disease-related outcome.
· Predictive factor	A predictive factor is a factor that provides information about the likely benefit of a patient from a specific treatment or therapeutic intervention; they help identifying patient subgroups that will (or will not) benefit from specific therapies.[2]
· Prognostic factor	A prognostic factor is a factor that provides information on the likely outcome or course of a disease in a patient; prognostic factors help predict the future course of the disease, regardless of the treatment received.
· Exposure and Outcome	Depending on the research questions, factors are designated as exposures or outcomes.<br>***Outcomes:*** Factors that are usually of clinical interest (e.g., disease status, death) used as endpoint in the analysis.<br>***Exposures:*** Factors that are assessed for their association or causal effect on outcomes, or for their ability to predict outcomes; exposures might be any type of a factor, but the word exposure often suggests that the factor can be eliminated.
**Prognostic value**	The ability of a prognostic factor to predict future outcomes, commonly measured by the widely used criteria of calibration, discrimination, and clinical utility.[3,4]
· Calibration	Calibration refers to the degree to which absolute risks or pure risks predicted from a prognostic model agree with the empirically observed risks.[5] Calibration plots are often presented to compare observed risks to predicted risks. A prediction model can lead to bad clinical decisions if it over- or under-estimates risk in some groups of patients or in the population as a whole.
· Discrimination	Discrimination refers to the ability to differentiate those patients at higher risk from those at lower risk.[5] It is typically measured by the area under the receiver operating characteristic curve (AUC) or the concordance (C) statistic and is interpreted as the probability that a randomly selected person with the outcome will have a higher predicted risk than a randomly selected person without the outcome.
· Prediction error	Prediction error is a measure of the discrepancy between observation and prediction. The Brier score, a measure of the overall prediction performance (or mean squared error), is a traditional measure for binary outcomes and can be evaluated over time (prediction error curve).[6,7]
· Clinical utility	For clinical implementation, it is also necessary to demonstrate that a prognostic factor or model effectively improves clinical utility or management. If the model is useful in clinical decision-making, it will improve the expected patient outcomes compared to standard of care and thereby increase the ratio of benefits-to-risks.[8,9]
**Study design**	In prognosis research, observational study designs are the usual choice, mainly because of ethical considerations, but also because of limited ability to manipulate prognostic factors.
· Prospective cohort study	Cohort of patients with a specific disease followed prospectively in time for the occurrence of the outcome(s) of interest; at the end of a study, occurrence of the outcome(s) of interest or death may or may not have been observed for participants (**Figure 2**).[10]<br>*Advantage:* Among the observational study designs, the prospective cohort study design may yield the highest quality data because the study population is well defined, standardized measurements and ascertainment procedures to obtain data including observation of the outcomes of interest.

	Disadvantage: expensive and time consuming
· Retrospective cohort study	In contrast to a prospective cohort study, the study population is retrospectively assembled from data records of patients who were previously diagnosed with the disease of interest and followed (**Figure 2**). Sources include previously established biospecimen or patient registries, public or commercial databases and electronic health records.<br>*Advantage:* readily available (baseline) data and biospecimens, one may not need to wait to observe the events of interest.<br>*Disadvantage:* potential issues defining the source population and a representative cohort, inability to gather high quality and complete baseline data, loss to follow-up, incomplete outcome ascertainment.<br>Note: STROBE prefers to avoid the term "retrospective cohort", given that many advantages and analyses are the same with prospective data collection. However, we acknowledge that the term persists in the peer-reviewed literature.
· Case-control study	In prognosis research, a case-control study compares patients with a particular disease who subsequently had the outcome event of interest (cases) with patients with the same disease who did not (controls), and it is characterized by a sampling based on outcome.[10] Exposure information is obtained retrospectively from cases and controls using questionnaire data or other data sources, such as medical records.<br>*Advantage:* especially suitable for outcomes of less frequency<br>*Disadvantage:* threat of non-representative selection of cases and controls, recall bias (i.e., differential recollection of exposure data from cases and controls)
· Nested case-control study<br>· Case-cohort study	Both these designs are based upon subsampling a cohort study. Such designs are usually used when certain questionnaire data items or laboratory data items are too costly or too difficult to obtain from all cohort participants.[10] They preserve the analytic options of the full cohort study, often with little loss in precision of estimates.<br>*Nested case-control design:* sampling of controls matched to cases on follow-up time and key factors (e.g., clinical).<br>*Case-cohort design:* inclusion of all cases and a random sample from the entire cohort.
**Types of data**	Each data item (factor) can assume values in a defined range of values. Different types require different handling in statistical analyses. Here the main types relevant in prognostic factor studies are explained:
· Discrete data	Factors that have a countable number of possible values/modalities.
· Binary data	Discrete factors that only can assume 2 values/modalities, e.g., yes or no for the presence of a disease; also called dichotomous.
· Categorical data	Discrete factors that can assume >2 values/modalities with either ordinal scale (i.e., natural order; e.g., disease stages) or nominal scale (e.g., ethnicities)
· Continuous data	Data items of this type can assume any value (i.e., real number) in a prespecified range, e.g., age ranging from 0 to 100 years.
· Time-to-event data	In cohort studies, the time from an origin, for example date of study entry or date of birth, to the occurrence of a predefined, incident event (i.e., outcome) is usually the outcome of interest. Time-to-event methodology can appropriately handle information from participants who are not observed to have the outcome of interest (i.e., censored without event) because of an earlier study closure, loss to follow-up, or occurrence of a competing event (see also *Survival methods*). If death is the outcome of interest, the term survival time is often used.
**Statistical methods**	The choice of statistical methods depends on the research question and data obtained.

· Statistical test	A statistical test is a rule for deciding whether to reject a statistical hypothesis. For example, if the hypothesis is that two groups have the same distribution of a measurement, a t-test or chi-square test might be used. If the statistical test exceeds a certain critical value, the hypothesis is rejected, and a p-value can be presented. Alternatively, a confidence interval for the parameter of interest can be constructed, and the hypothesis rejected if the confidence interval excludes the hypothesized parameter value.
· Regression model	In regression analysis, models are fitted to estimate the dependence of an outcome variable (dependent variable) on one or more explanatory variables (independent variables: e.g., other prognostic factors, confounding factors).[11,12] There are different uses for regression models including estimating associations with the outcome or predicting the outcome. Depending on the outcome data, different regression models are available such as the logistic regression model for binary outcome data.[13]
· Survival methods	For the analysis of time-to-event data, survival methods allow to appropriately deal with censored observations. They include methods for the regression of the outcome hazard on independent variables (e.g., Cox proportional hazard regression), estimation of the survival curve (1 minus the distribution function of the time-to-event), and estimation of pure risk or absolute risk (also called estimated cumulative incidence).[11,14] Specific methods are available to estimate absolute risk in the presence of competing events (e.g., cause-specific hazard models or Fine & Gray proportional sub-distribution hazard model).
**Estimate / estimand**	The estimand is the measure of interest (e.g., the effect of an exposure on an outcome in the target population) one wants to estimate in a study. The estimate is the observed statistical quantity (numerical value / result) of the estimand.
· Absolute risk	The absolute risk (also crude risk or cumulative incidence) is the probability that the event will occur within a specified period of time. Survival curves as a function of time *t* describe the probability of not having the event (1 - absolute risk) over time. Absolute risk is also used to denote the probability that an event will occur within a specified time period accounting for the presence of competing risks.
· Pure risk	The pure risk is the probability that the event will occur within a specified period of time presuming the absence of competing events. If competing events are present, the pure risk estimate is greater than the absolute risk estimate that takes competing events into account. Absolute risk that takes competing risks into account is of greater interest than pure risk for prognosis research, because patients usually face unavoidable competing risks.
· Relative risk	The relative risk is the ratio of the probability of an outcome of one group (e.g., exposed participants) to the probability of the outcome in another group (e.g., unexposed participants).
· Relative odds / odds ratio	The estimate of the relative odds (or odds ratio) in cohort studies is the ratio of the odds of a binary outcome in exposed persons to the odds in unexposed persons. In case-control studies, one estimates the ratio of the odds of exposure (probability of exposure happening divided by the probability of not happening) in those with the outcome (cases) to the odds of exposure in those without the outcome (controls).[13] The odds ratio can be estimated consistently with both study designs. When the outcome is rare (rare disease assumption), the odds ratio approximates the relative risk quite well.
· Relative hazard / hazard ratio	In the analysis of time-to-event data, the instantaneous hazard rate of the outcome event at time *t* can be estimated. The association between the factor of interest and survival can be expressed as the ratio of the hazards (hazard ratio) in those with and without the factor (if binary) or per unit increase (if continuous).[11]

References

1. Califf RM. Biomarker definitions and their applications. *Exp Biol Med Maywood NJ*. 2018;243(3):213-221. doi:10.1177/1535370217750088

2. McShane LM, Hayes DF. Publication of tumor marker research results: the necessity for complete and transparent reporting. *J Clin Oncol Off J Am Soc Clin Oncol*. 2012;30(34):4223-4232. doi:10.1200/JCO.2012.42.6858

3. Steyerberg EW. *Clinical Prediction Models: A Practical Approach to Development, Validation, and Updating*. Springer; 2009.

4. Pfeiffer RM, Gail MH. *Absolute Risk: Methods and Applications in Clinical Management and Public Health*. CRC Press Taylor & Francis; 2018.

5. Alba AC, Agoritsas T, Walsh M, et al. Discrimination and Calibration of Clinical Prediction Models: Users' Guides to the Medical Literature. *JAMA*. 2017;318(14):1377. doi:10.1001/jama.2017.12126

6. Steyerberg EW, Vickers AJ, Cook NR, et al. Assessing the performance of prediction models: a framework for traditional and novel measures. *Epidemiol Camb Mass*. 2010;21(1):128-138. doi:10.1097/EDE.0b013e3181c30fb2

7. Schoop R, Beyersmann J, Schumacher M, Binder H. Quantifying the predictive accuracy of time-to-event models in the presence of competing risks. *Biom J Biom Z*. 2011;53(1):88-112. doi:10.1002/bimj.201000073

8. Steyerberg EW, Moons KGM, van der Windt DA, et al. Prognosis Research Strategy (PROGRESS) 3: prognostic model research. *PLoS Med*. 2013;10(2):e1001381. doi:10.1371/journal.pmed.1001381

9. Sachs MC, Sjölander A, Gabriel EE. Aim for Clinical Utility, Not Just Predictive Accuracy. *Epidemiology*. 2020;31(3):359-364. doi:10.1097/EDE.0000000000001173

10. Gail MH, Altman DG, Cadarette SM, et al. Design choices for observational studies of the effect of exposure on disease incidence. *BMJ Open*. 2019;9(12):e031031. doi:10.1136/bmjopen-2019-031031

11. Harrell FE. *Regression Modeling Strategies: With Applications to Linear Models, Logistic and Ordinal Regression, and Survival Analysis*. Second edition. Springer; 2015.

12. Greenland S, Morgenstern H. Confounding in health research. *Annu Rev Public Health*. 2001;22:189-212. doi:10.1146/annurev.publhealth.22.1.189

13. Hosmer DW, Lemeshow S, Sturdivant RX. *Applied Logistic Regression*. Third edition. Wiley; 2013.

14. Perera M, Dwivedi AK. Statistical issues and methods in designing and analyzing survival studies. *Cancer Rep Hoboken NJ*. 2020;3(4):e1176. doi:10.1002/cnr2.1176
