# Supplementary material for: Design aspects for prognostic factor studies
Source: BMJ Open. 2025 Aug 31;15(8):e095065. doi: 10.1136/bmjopen-2024-095065 (PMC12406931; doi:10.1136/bmjopen-2024-095065)
Supplement: Supplementary file 2 [file bmjopen-2024-095065.reviewer_comments.pdf]

PEER REVIEW HISTORY

BMJ Open publishes all reviews undertaken for accepted manuscripts. Reviewers are asked to complete a checklist review form and are provided with free text boxes to elaborate on their assessment. These free text comments are reproduced below.

ARTICLE DETAILS

Title (Provisional)

Design Aspects for Prognostic Factor Studies

Authors

Sekula, Peggy; Steinbrenner, Inga; Schultheiss , Ulla T; Valveny, Neus; Rebora, Paola; Halabi, Susan; Cadarette, Suzanne; Riley, Richard; Collins, Gary Stephen; Sauerbrei, Wilhelm F; Gail, Mitchell; STRATOS initiative, for topic group 5 of the

---

VERSION 1 - REVIEW

**Reviewer**	**1**
**Name**	**Holmberg, Christopher**
**Affiliation**	**University of Gothenburg Sahlgrenska Academy, Health and Care Sciences**
**Date**	**05-Apr-2025**
**COI**	**None**

Thank you for allowing me to review this manuscript. The article is timely and relevant. I understand that the author(s) are interested in raising awareness of the specific issues surrounding study design in prognostic studies. They state that they are affiliated with the "TG5: study design" of the STRATOS initiative.

The topic of study design in prognostic research has been addressed previously, in particular through existing guidelines and recommendations (e.g. REMARK guidelines, PROGRESS framework). While it does not introduce novel methods, it has the potential to reach novice researchers and translate statistical concepts into accessible guidance.

However, I have a few comments that I would like the author(s) to address:

Given the growing importance of "omics" data in prognosis research, it is a limitation that the manuscript explicitly excludes these studies. While this decision was mentioned, even a brief methodological note on the integration of high-dimensional data would enhance the value of the manuscript.

While the manuscript conceptually describes statistical approaches, it does not provide concrete computational examples or reference software packages. Providing such resources

would improve its utility for researchers applying these methods in practice. This is particularly important given that this is the audience they are addressing.

I have some concerns about the statistical depth. While basic concepts are covered (e.g., confounding, power), advanced topics such as dealing with continuous variables (other than "try not to categorize them for data loss purposes") or mentioning machine learning approaches are omitted. While I understand that the focus is on single factor and binary outcomes, I would still like to see some elaboration on these aspects in terms of design. For example, how do you deal with these variables if they are potential confounders? And a brief mention of machine learning approaches for large datasets.

Overall, this manuscript provides valuable, practical guidance for improving prognostic factor studies, in keeping with BMJ Open's mission to improve research quality and clinical relevance. While not entirely novel, its synthesis of existing knowledge in an accessible format makes it a timely contribution. I encourage the author(s) to resubmit a revised version.

**Reviewer**	**2**
**Name**	**Tortosa , Fernando**
**Affiliation**	**Pan American Health Organization, EIH**
**Date**	**17-Apr-2025**
**COI**	**None**

General assessment

This manuscript addresses essential considerations in the design of prognostic factor studies and provides a useful overview for a broad audience, especially non-specialist readers. It is clearly written, methodologically sound, and relevant to the aims of BMJ Open and its readership. The structured guidance it offers is highly valuable, particularly its focus on cohort studies, appropriate outcome selection, sample size considerations, and protocol development.

However, I believe the manuscript would benefit from the addition of a few important considerations that would improve its completeness, broaden its scope, and increase its practical applicability across diverse settings and diseases.

The manuscript is well structured and written in an accessible manner.

It is strongly aligned with relevant methodological initiatives such as STRATOS, PROGRESS, and REMARK.

It includes highly useful clarifications on estimands, definitions of outcome, and timing of measurements.

It provides concrete examples and explanatory figures/tables to support interpretation.

Suggestions for improvement

Perspective focused on cohort studies

While the article offers comprehensive guidance for prospective cohort designs, the focus limits generalizability. It would be helpful to more explicitly state that some of the recommendations may not directly apply to other designs (e.g., nested case-control, retrospective cohorts, or real-world data studies), or require adaptation.

Prevalence and predictive value

One important omission is a discussion on how prevalence of the prognostic factor affects its predictive value. Especially in clinical practice, even a factor with strong association may have low utility if the condition is rare or the factor is highly prevalent. I recommend citing the work of Faruotan et al., which discusses adjustment of prognostic value to prevalence and explores how to derive clinically meaningful predictions beyond relative associations.

Validation – need for operational clarity

Although internal and external validation are rightly emphasized, the manuscript could be strengthened by providing clearer operational criteria or examples on how external validation should be conducted and reported (e.g., minimum requirements such as same outcome definitions, different populations, blinded measurement).

Multiple small studies and evidence synthesis

In rare diseases or low-incidence outcomes, prognostic evidence may come from multiple small or heterogeneous studies, rather than a single large prospective cohort. The manuscript would benefit from acknowledging the value of systematic reviews and meta-analyses of prognostic factor studies (e.g., Riley et al., BMJ 2019) and how such syntheses can support model building or decision-making.

Visual summary for design guidance

A schematic summary (e.g., decision flowchart or checklist) illustrating the key steps in planning a prognostic factor study would significantly enhance the manuscript's utility, especially for applied researchers.

Discussion of known gaps

Including a short final section or paragraph explicitly identifying knowledge gaps (e.g., how to manage poor-quality data in EHR-based prognostic studies, how to prioritize factors for external validation, or reporting standards in low-resource settings) would broaden the appeal and underscore the urgency for future research and consensus.

Editorial recommendations

Expand the discussion on external validation and its implementation.

Include a brief commentary on limitations of studies in rare diseases, and the role of evidence synthesis or collaborative data approaches.

Consider incorporating additional references, such as:

Riley et al., BMJ 2019; A guide to systematic review and meta-analysis of prognostic factor studies.

Faruotan et al., on prevalence-adjusted predictive value.

If possible, include a supplemental figure or checklist to aid application of design principles.

This is a well-conceived manuscript that addresses a timely and important methodological topic. It has the potential to serve as a reference for clinicians, epidemiologists, and trialists interested in prognostic factor research. With the suggested additions, it will better reflect the breadth of challenges in real-world research and contribute even more meaningfully to the improvement of prognostic research standards.

VERSION 1 - AUTHOR RESPONSE

Reviewer: 1
Dr. Christopher Holmberg, University of Gothenburg Sahlgrenska Academy

Thank you for allowing me to review this manuscript. The article is timely and relevant. I understand that the author(s) are interested in raising awareness of the specific issues surrounding study design in prognostic studies. They state that they are affiliated with the "TG5: study design" of the STRATOS initiative.

The topic of study design in prognostic research has been addressed previously, in particular through existing guidelines and recommendations (e.g. REMARK guidelines, PROGRESS framework). While it does not introduce novel methods, it has the potential to reach novice researchers and translate statistical concepts into accessible guidance.

Response: We thank Dr. Holmberg for taking time to assess our contribution on prognostic factor studies. It was indeed our intention to add this introduction to the field for less experienced researchers and non-statisticians.

However, I have a few comments that I would like the author(s) to address:

Given the growing importance of "omics" data in prognosis research, it is a limitation that the manuscript explicitly excludes these studies. While this decision was mentioned, even a brief methodological note on the integration of high-dimensional data would enhance the value of the manuscript.

Response: We agree that the analysis of high-dimensional omics data is becoming increasingly important. For this reason, we have relaxed our restriction by linking our focus on analyzing a single factor to the analysis high-dimensional data. In the chapter ‘Statistical methods’ (second to last paragraph, p.16), we have included a separate paragraph on alternative methods. We mention dimension reduction techniques including penalized regression (e.g., LASSO) and machine learning/artificial intelligence approaches (e.g., random forest, neural networks).
In consequence, further small changes on text were made in the ‘Abstract’ (p.3), ‘Introduction’ (last paragraph, p.5), ‘Aims of prognostic factor studies’ (2nd paragraph, p.6) and ‘Summary’ (p.19).

While the manuscript conceptually describes statistical approaches, it does not provide concrete computational examples or reference software packages. Providing such resources would improve its utility for researchers applying these methods in practice. This is particularly important given that this is the audience they are addressing.

Response: We thank the Dr. Holmberg for this idea. However, as there are many different statistical software packages (e.g., STATA, SAS, R, SPSS, Prism, Mathematica …) and prognostic factor studies have different objectives requiring different steps of statistical analysis, this is beyond the scope of this manuscript, but it would be an interesting follow-up idea – for example, to provide a blueprint for a specific setting.

I have some concerns about the statistical depth. While basic concepts are covered (e.g., confounding, power), advanced topics such as dealing with continuous variables (other than "try not to categorize them for data loss purposes") or mentioning machine learning approaches are omitted. While I understand that the focus is on single factor and binary outcomes, I would still like to see some elaboration on these aspects in terms of design. For example, how do you deal with these variables if they are potential confounders? And a brief mention of machine learning approaches for large datasets.

Response: Thank you for this comment and we are happy to include additional information. We elaborate on the topics of dealing with continuous variables and specific methods for omics data in two new paragraphs added to 'Statistical methods' (5th paragraph, p.15).

Overall, this manuscript provides valuable, practical guidance for improving prognostic factor studies, in keeping with BMJ Open's mission to improve research quality and clinical relevance. While not entirely novel, its synthesis of existing knowledge in an accessible format makes it a timely contribution. I encourage the author(s) to resubmit a revised version.

Response: We thank the Dr. Holmberg for his valuable comments and hope that our additions are to his satisfaction.

Reviewer: 2
Dr. Fernando Tortosa, Pan American Health Organization

General assessment
This manuscript addresses essential considerations in the design of prognostic factor studies and provides a useful overview for a broad audience, especially non-specialist readers. It is clearly written, methodologically sound, and relevant to the aims of BMJ Open and its readership. The structured guidance it offers is highly valuable, particularly its focus on cohort studies, appropriate outcome selection, sample size considerations, and protocol development.

However, I believe the manuscript would benefit from the addition of a few important considerations that would improve its completeness, broaden its scope, and increase its practical applicability across diverse settings and diseases.

The manuscript is well structured and written in an accessible manner.

It is strongly aligned with relevant methodological initiatives such as STRATOS, PROGRESS, and REMARK.

It includes highly useful clarifications on estimands, definitions of outcome, and timing of measurements.

It provides concrete examples and explanatory figures/tables to support interpretation.

Response: We thank Dr. Tortosa for his good assessment and suggestions for improvement.

Suggestions for improvement

Perspective focused on cohort studies
While the article offers comprehensive guidance for prospective cohort designs, the focus limits generalizability. It would be helpful to more explicitly state that some of the recommendations may not directly apply to other designs (e.g., nested case-control, retrospective cohorts, or real-world data studies), or require adaptation.

Response: We agree with the Reviewer that the manuscript's focus is on cohort studies and cannot cover every single aspect for whatever design a researcher would like to implement. For this reason we extended our statement on the limitations in 'Summary' of the manuscript (p.19) expressing this circumstance.

Prevalence and predictive value
One important omission is a discussion on how prevalence of the prognostic factor affects its predictive value. Especially in clinical practice, even a factor with strong association may have low utility if the condition is rare or the factor is highly prevalent. I recommend citing the work of Faruotan et al., which discusses adjustment of prognostic value to prevalence and explores how to derive clinically meaningful predictions beyond relative associations.

Response: Thank you for this comment. In the manuscript we inserted additional remarks accordingly.
In chapter 'Aims of prognostic factor studies', we discuss in the 3rd paragraph (p.6) the necessity of providing absolute risks. In this context, we mention that often large association effects are needed and that the prognostic value and eventually its clinical utility depends on the prevalence/variability of factor and the incidence of outcome. At this place we also inserted the requested citation (Ref #41: *Foroutan et al. Calculation of absolute risk for important outcomes in patients with and without a prognostic factor of interest. J Clin Epidemiol. 2020*).
Furthermore, we pick up the necessity of providing absolute risks in the chapter 'Statistical Methods' (4th paragraph, p.15) by stating that regression models that foremost provide relative measures of association can also be used to derive absolute measures, though of external data are required in case of a case-control study.
Finally, we also mention that the prevalence of the factor is one of the ingredients for calculation of sample size/power in the chapter 'Sample size and power of a prognostic factor study' (2nd paragraph, p.17).

Validation – need for operational clarity
Although internal and external validation are rightly emphasized, the manuscript could be strengthened by providing clearer operational criteria or examples on how external validation should be conducted and reported (e.g., minimum requirements such as same outcome

definitions, different populations, blinded measurement).

Response: We agree with Dr. Tortosa that validation is an important topic. We have therefore expanded this topic at different places. First of all, we updated the respective entries in our Glossary (previously **Box 2**, now **Supplemental Information**) to provide more specific definitions. In the chapter 'Aims of prognostic factor studies' (4th paragraph, p.6), we included external validation as a possible other aim of prognostic factor studies. In the chapter 'Statistical methods' (last paragraph, p.16), we provide reference for methods when aiming on validation. In the chapter 'Study protocol and reporting' (3rd paragraph, p.18), we explicitly added the TRIPOD-AI guideline. Though this guideline is aimed to support prognostic model research when aiming to develop and validate prognostic models, many aspects apply to prognostic factor studies as well. Finally, we picked up the topic of validation in our 'Summary' statement (p.19). Though many additional aspects could be mentioned, we believe that this is beyond the scope of this particular manuscript.

Multiple small studies and evidence synthesis
In rare diseases or low-incidence outcomes, prognostic evidence may come from multiple small or heterogeneous studies, rather than a single large prospective cohort. The manuscript would benefit from acknowledging the value of systematic reviews and meta-analyses of prognostic factor studies (e.g., Riley et al., BMJ 2019) and how such syntheses can support model building or decision-making.

Response: Thank you for this comment. To put emphasis on the use of evidence synthesis, we expanded the chapter 'Aims of prognostic factor studies' (next to last paragraph, p.7) and the chapter 'Sample size and power of a prognostic factor study' (4th paragraph, p.17). In this context, we also stress the importance of a meta-analysis using individual participant data (IPD). Riley et al. BMJ 2019 is referenced in both paragraphs (Ref #29). In the end, we even mention this topic now in our 'Summary' statement (p.19).

Visual summary for design guidance
A schematic summary (e.g., decision flowchart or checklist) illustrating the key steps in planning a prognostic factor study would significantly enhance the manuscript's utility, especially for applied researchers.

Response: Table 2 (p.34) contains a summarized overview on different aspect to consider when designing a prognostic factor study. Though completeness of this table can be debated, this table may serve as a check list. Since aspects are related, a schematic illustration of a flowchart is difficult. Still, to emphasize dependencies, we added a new figure (**Figure 3**, p.36) to the manuscript. **Table 2** was revised at this point as well to harmonize both display items. Both are referenced in 'Study protocol and reporting' (2nd paragraph, p.18). A concluding statement was added to our 'Summary' statement (p.20).

Discussion of known gaps
Including a short final section or paragraph explicitly identifying knowledge gaps (e.g., how to manage poor-quality data in EHR-based prognostic studies, how to prioritize factors for external validation, or reporting standards in low-resource settings) would broaden the appeal and underscore the urgency for future research and consensus.

Response: We agree with Dr. Tortosa that there is no complete answer to every issue or

problem that might exist. Also new data sources or lab techniques may add new items to the list of open issues. To emphasize that there are still also methodological knowledge gaps, we have updated the 'Summary' statement (p.20) accordingly.

VERSION 2 - REVIEW

**Reviewer**	**1**
**Name**	**Holmberg, Christopher**
**Affiliation**	**University of Gothenburg Sahlgrenska Academy, Health and Care Sciences**
**Date**	**04-Jul-2025**
**COI**	

The author(s) have satisfactorily addressed my previous comments. Thank you.

**Reviewer**	**2**
**Name**	**Tortosa , Fernando**
**Affiliation**	**Pan American Health Organization, EIH**
**Date**	**07-Jul-2025**
**COI**	

The revised manuscript provides an improved and comprehensive overview of the methodological principles underpinning prognostic factor studies. The authors have successfully addressed most prior suggestions, particularly by expanding the discussion on the role of systematic reviews and meta-analyses (citing Riley et al.) and by emphasizing the need for operational clarity in external validation and multi-study synthesis. I appreciate the enhanced discussion of how prevalence and clinical context affect the real-world utility of prognostic factors; this addition improves the practical guidance for researchers and clinicians. However, the manuscript would benefit from an explicit reference to established methodological work (such as Foroutan et al., BMJ 2020) which offers a transparent framework for adjusting the predictive value of prognostic factors by prevalence—a key consideration for interpretation and clinical translation. The overall structure and practical focus of the manuscript, including the checklist and figures, make it a valuable resource for both applied and methodological audiences. I recommend acceptance after the addition of a reference and a brief note about how prevalence-adjusted metrics can inform individual risk communication and decision-making.

VERSION 2 - AUTHOR RESPONSE

Reviewer: 1 Dr. Christopher Holmberg, University of Gothenburg Sahlgrenska Academy

The author(s) have satisfactorily addressed my previous comments. Thank you.

Response: We thank Dr. Holmberg for his time and support to improve the manuscript.

Reviewer: 2 Dr. Fernando Tortosa , Pan American Health Organization

The revised manuscript provides an improved and comprehensive overview of the methodological principles underpinning prognostic factor studies. The authors have successfully addressed most prior suggestions, particularly by expanding the discussion on the role of systematic reviews and meta- analyses (citing Riley et al.) and by emphasizing the need for operational clarity in external validation and multistudy synthesis. I appreciate the enhanced discussion of how prevalence and clinical context affect the realworld utility of prognostic factors; this addition improves the practical guidance for researchers and clinicians. However, the manuscript would benefit from an explicit reference to established methodological work (such as Foroutan et al., BMJ 2020) which offers a transparent framework for adjusting the predictive value of prognostic factors by prevalence—a key consideration for interpretation and clinical translation.

The overall structure and practical focus of the manuscript, including the checklist and figures, make it a valuable resource for both applied and methodological audiences. I recommend acceptance after the addition of a reference and a brief note about how prevalence-adjusted metrics can inform individual risk communication and decision-making.

Response: We thank Dr. Tortosa. We agree that the prevalence of the prognostic factor influences the predictive values of the prognostic factor and possibly also of the prognostic model that includes this factor, albeit more indirectly than the prevalence of the outcome itself. However, the analysis of predictive values becomes particularly relevant when the clinical utility of a prognostic model is in the primary focus of research. It therefore represents an important aspect of prognostic model research. Since the focus of this contribution is on prognostic factor research, we have addressed this topic only briefly on page 6 (3rd paragraph, yellow background). In order to maintain the intended focus of this contribution, we have chosen not to elaborate on this aspect further.
